# Glycine *N*‐Acyltransferase Deficiency due to a Homozygous Nonsense Variant in the *GLYAT*: A Novel Inborn Error of Metabolism

**DOI:** 10.1002/jmd2.70032

**Published:** 2025-07-29

**Authors:** Mona Nourbakhsh, Mohammad Miryounesi, Ali Tale, Parvaneh Karimzadeh, Hossein Sadeghi, Mohammad‐Reza Ghasemi, Nasrin Alipour, Elham Pourbakhtyaran, Nakisa Hooman, Maryam Razzaghy‐Azar, Mitra Nourbakhsh, Lil Klaas, Daniel Schulke, Jörn Oliver Sass

**Affiliations:** ^1^ Aliasghar Clinical Research Development Center, Department of Pediatrics, School of Medicine Iran University of Medical Sciences Tehran Iran; ^2^ Department of Medical Genetics, School of Medicine Shahid Beheshti University of Medical Sciences Tehran Iran; ^3^ Metabolic Disorders Research Center, Endocrinology and Metabolism Molecular‐Cellular Sciences Institute Tehran University of Medical Sciences Tehran Iran; ^4^ Department of Pediatric Neurology, Pediatric Neurology Research Center, Research Institute for Children's Health Shahid Beheshti University of Medical Sciences Tehran Iran; ^5^ Genomic Research Center Shahid Beheshti University of Medical Sciences Tehran Iran; ^6^ Finetech in Medicine Research Center Iran University of Medical Sciences Tehran Iran; ^7^ Department of Clinical Biochemistry, School of Medicine Iran University of Medical Sciences Tehran Iran; ^8^ Research Group Inborn Errors of Metabolism, Department of Natural Sciences & Institute for Functional Gene Analytics (IFGA) Bonn‐Rhein‐Sieg University of Applied Sciences Rheinbach Germany

**Keywords:** glycine *N*‐acyltransferase deficiency, hyperglycinemia, neurometabolic disorder

## Abstract

The enzyme glycine *N*‐acyltransferase (GLYAT) plays a crucial role in detoxifying both xenobiotic and endogenous compounds that contain a carboxylic acid group, such as benzoic acid. Data on the impact of human GLYAT on the glycine conjugation pathway is limited and difficult to determine. In this study, we present a 5.7‐year‐old girl with gross motor delay first noticed at age 5 months and speech delay evident at the time of diagnosis. To the best of our knowledge, no case of GLYAT enzyme deficiency has been reported to date. Whole exome sequencing (WES) identified a homozygous nonsense variant (NM_201648.3: c.322C>T: p.(Q108Ter)) in the *GLYAT* that abolished GLYAT activity in vitro. The detected variant was confirmed by Sanger sequencing. The patient was treated with pantothenic acid and a mitochondrial cocktail consisting of coenzyme Q10, vitamins B1, B2, B6, B12, C, folate, and carnitine, together with a low‐protein diet, which led to the alleviation of edema and hypotonia and an improvement in her motor function and social interactions. Her serum glycine level was also normalized. This case identifies a novel homozygous nonsense variant in the *GLYAT*, leading to glycine *N*‐acyltransferase enzyme deficiency and associated developmental delays.

**Summary**

- This article presents the first reported case of GLYAT enzyme deficiency caused by a novel homozygous nonsense variant in the *GLYAT*.

## 1 Introduction

Glycine *N*‐acyltransferase (GLYAT) (EC:2.3.1.13) is a monomeric detoxification enzyme found in the mitochondrial matrix of mammalian liver and kidney cells [1]. This enzyme has an apparent molecular mass of 30 kD and is encoded by *GLYAT* (gene ID: 10249) [2]. This mitochondrial enzyme conjugates glycine with acyl‐coenzyme A (CoA) residues; therefore, it has an important role in detoxifying endogenous and exogenous substrates, acting on their CoA derivatives. It has substrate specificity for benzoyl‐CoA, salicyl‐CoA, isovaleryl‐CoA, and octanoyl‐CoA, with the lowest Km for benzoyl‐CoA [2, 3]. GLYAT conjugates benzoyl‐CoA to form hippuric acid, releasing coenzyme A (CoASH) [4]. Glycine conjugation is also an effective way to eliminate the end products of phenylpropionate metabolism [5]. Removing CoA from its derivatives helps to recycle CoA and prevents its sequestration [4].

GLYAT performs xenobiotic metabolism using glycine. Therefore, this enzyme indirectly maintains glycine balance. Glycine is a neurotransmitter in the central nervous system (CNS) [6]. Thus, maintaining a balance between glycine biosynthesis and clearance is critical for properly regulating its excitatory and inhibitory neurological functions. Glycine is cleared from the body through both metabolic degradation and the glycine conjugation pathway, which involves scavenging by benzoic acid and excretion as hippurate [6]. Deficiency of the glycine cleavage enzyme system, leading to significantly elevated glycine levels, causes neonatal nonketotic hyperglycinemia (NKH), an autosomal recessive disorder characterized by severe neurological symptoms and early mortality [7]. However, defects in the glycine conjugation pathway, an alternative mechanism for glycine clearance, are also proposed to result in a mild accumulation of glycine and associated neurological effects [5, 6]. Here we present the first case of impaired glycine conjugation due to a homozygous variant in the *GLYAT* associated with transient hyperglycinemia.

## 2 Materials, Methods and Results

### 2.1 Case Presentation

The study was exempt from IRB oversight due to the retrospective data and patient deidentification. Informed consent was obtained from the patient's guardian.

The patient is a 5‐year‐ and 8‐month‐old girl who was referred at 10 months of age due to developmental delay. She was born at 38 weeks of gestation via elective cesarean section, with an Apgar score of 8/10. Her birth weight was 3.55 kg, and her head circumference was 35 cm. She had a history of neonatal jaundice, requiring phototherapy for a bilirubin level of 18 mg/dL. She is the second child of first‐cousin parents. The mother was treated for hypothyroidism and had gestational diabetes during the pregnancy. There was no family history of neurological disorders, and the patient had no history of seizures.

At 5 months, her developmental delay became apparent with an inability to hold her head up and mild hypotonia. She exhibited delays in neurodevelopmental milestones, rolling over at 9 months, sitting at 12 months, and crawling at 14 months. By the age of 5 years and 8 months, she was only able to speak 3–4 words but could recognize objects and follow simple commands. She was unable to sit but remained attentive to her parents.

On physical examination at 10 months of age, her weight was 7 kg (below the 3rd percentile), and her height was 66 cm (at the 3rd percentile), according to CDC 2000 growth charts. The anterior fontanel measured 1.5 × 1.5 cm and was open. There was no organomegaly, and no abnormalities were found in her heart, lungs, limbs, or skull.

At 10 months of age, tests for routine biochemical analytes, enzymes, the acylcarnitine profile, and urine organic acids were performed, with all results found to be normal. Chromatography of plasma and urine amino acids was performed using high‐performance liquid chromatography (HPLC) (Agilent 1100) and thin‐layer chromatography of urine sugars, which did not reveal any abnormalities. Serum homocysteine, lactate, pyruvate, and ammonia levels were also normal.

At 2 years of age, she continued to experience difficulties with walking and speaking. As a result, plasma and urine amino acid chromatography were repeated. The results showed a mild increase in plasma glycine, which tended to rise over time (Table 1). Glycine in CSF was 8 μmol/L (≤ 12 μmol/L), and the CSF/plasma glycine ratio was 0.01 (normal < 0.04). Urine glycine was elevated at 1836 μmol/L (normal range: 23–413 μmol/L). There was no evidence of acidosis or ketosis. Based on an initial diagnosis of non‐ketotic hyperglycinemia, a low‐glycine diet was implemented. Sodium benzoate was also prescribed at the dose of 250 mg/kg, which led to difficulty concentrating, unusual hand movements, and a seizure; so, it was discontinued. Phenobarbital was started to control the seizures, and since the seizures seemed to be due to sodium benzoate, it was not prescribed again. During the physical and neurological examination, she was alert and exhibited horizontal nystagmus. She could sit independently but was unable to stand or walk without assistance. Her deep tendon reflexes (DTRs) were normal. Electroencephalography (EEG) showed moderate abnormalities with bilateral paroxysmal epileptic discharges. Brain magnetic resonance imaging (MRI) was performed, which returned normal results. Auditory brainstem response (ABR) testing revealed a slight sensorineural hearing loss.

**TABLE 1:** Serum glycine levels at various time points.

	Time point	Glycine concentration	Remarks
1	At admission	430 μmol/L	
2	Two weeks after admission	581 μmol/L	
3	Four weeks after admission	696 μmol/L	A genetic study was done at this time point.
4	Four months after the genetic study	759 μmol/L	A low‐protein diet was administered during this period. Treatment was initiated at this time.
**5**	Two months after the initiation of treatment	507 μmol/L	
**6**	Three months after the initiation of treatment	230 μmol/L	

At 2 years and 10 months, she was readmitted to the hospital due to generalized edema. At that time, she was unable to stand due to muscle weakness. She also exhibited autistic features, such as a lack of social communication and interaction, no eye contact, and no reply to simple questions. The Bayley Scales of Infant and Toddler Development were used, which generally assess cognitive, language, fine and gross motor, and social–emotional abilities [8].

Laboratory tests revealed significant proteinuria (+4) and hematuria (+3), with a total protein level of 2.72 g/L (normal range: 5.1–8) and low plasma albumin at 1.16 g/L (normal range: 3.8–5.4). Thyroid function tests indicated hypothyroidism, with total T4 at 2.979 μg/dL (normal range: 5–13) and Thyroid stimulating hormone at 10.137 mIU/mL (normal range: 0.5–5.5), for which she was treated with levothyroxine. Her serum lipid levels were significantly elevated, with total cholesterol at 459 mg/dL (normal: 120–216 mg/dL), low‐density lipoprotein (LDL) at 345 mg/dL (normal range for age < 110), and triglycerides at 292 mg/dL (normal: 44–197 mg/dL).

### 2.2 Genetic Analysis

**FIGURE 1:**
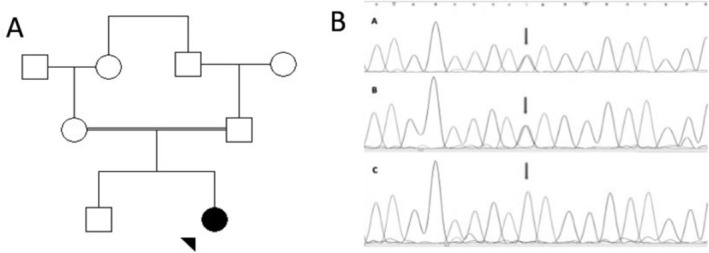
A. Pedigree of the family with glycine *N*‐acyltransferase deficiency. The arrow represents the proband, and open symbols represent normal individuals. B. Electropherogram for exon 5 of *GLYAT*. The arrow symbol indicates the NM_201648.3: C.322C>T in the father (A), mother (B), and affected girl (C).

The DNA was extracted from the blood sample using the standard salting‐out method. Whole exome sequencing (WES) was performed for the proband (Figure 1A). Raw data from WES of the patients and the parents are included as Supplementary Files 1–3. A DNA sample was sequenced on an Illumina HiSeq 4000 machine (Illumina Inc., USA). Rare variants with a minor allele frequency (MAF) < 0.1%, including nonsense, synonymous, nonsynonymous, splice site, insertion, and deletions, were selected for interpretation. The results showed a homozygous variant in the *GLYAT* (NM_201648.3: c.322C>T: p.(Q108Ter)). This gene has 6 exons and 296 amino acids. This variant leads to a premature stop codon and loss of amino acids 105–296. *GLYAT* variant characteristics are shown in Table 2. We also amplified exon 5 of the *GLYAT* with specific primers designed by Gene Runner software (version 3.05). Sanger sequencing was performed to confirm the variant in the affected girl. Furthermore, Sanger sequencing was performed on the parents of the proband (Supporting Information), and the variant was found to be segregated, and the parents were heterozygous for this variant (Figure 1B).

**TABLE 2:** *GLYAT* gene variant (NM_201648.3: C.322C>T) characteristics. This homozygous mutation leads to a stop codon and causes an early protein truncation.

Gene & transcript	Variant	Associated disease	Zygosity	dbSNPrsID	ACMG classification
*GLYAT*; NM_201648.3	Exon 5; c.322C>T:p.Q108X	Glycine *N*‐acyltransferase deficiency	Homozygous	rs201398923	VUS

*Abbreviations*: ACMG, American College of Medical Genetics and Genomics; VUS, Variant of Uncertain Significance.

### 2.3 Protein Overexpression, Purification, and Partial Characterization

**FIGURE 2:**
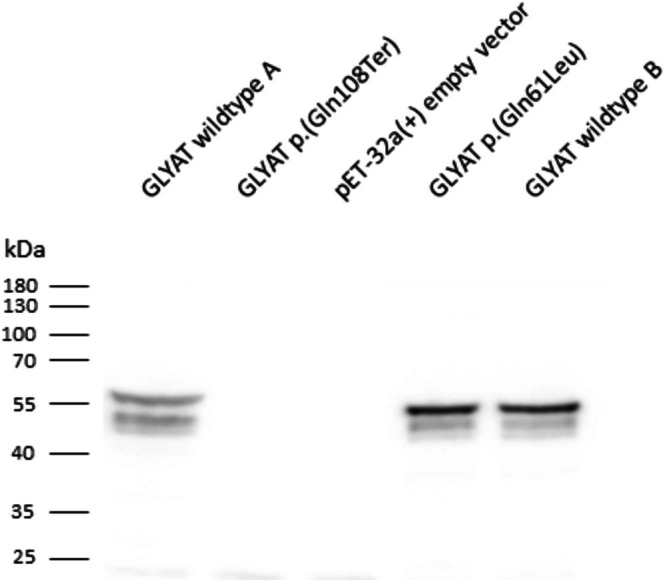
Immunoblot analysis of GLYAT protein after overexpression in *E. coli* Origami 2 (DE3) cells using a pET‐32a(+) vector providing a thioredoxin (Trx)‐tag and His‐tags. Homogenate supernatants (25 μg proteins), collected from bacterial lysates after centrifugation at 14 000 g and 4°C for 25 min, and His‐tag purified protein (2.5 μg protein each) were subjected to SDS‐PAGE (12%) followed by Western blot analysis. Anti‐GLYAT rabbit antibody (ThermoFisher, #PA5‐48504, 1:1000 in 5% skim milk in TBS‐T) was used. The secondary antibody was an anti‐rabbit IgG HRP‐linked donkey antibody (Cytiva, NA934). wt = wild type, homogenate = supernatant after centrifugation, purified = protein after His‐tag‐based purification.

**FIGURE 3:**
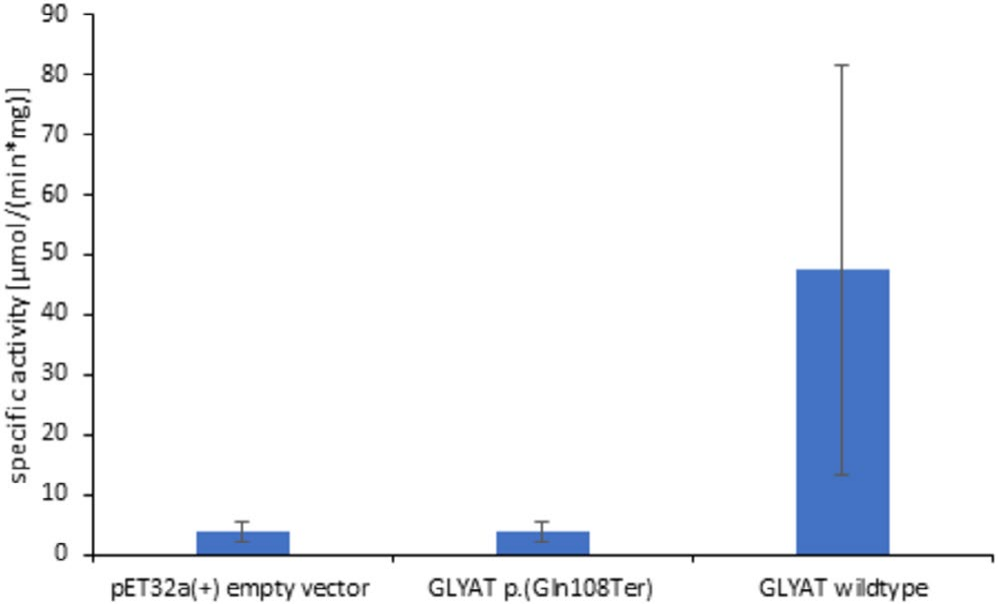
GLYAT enzyme activity determined after His‐tag‐based purification (see legend Figure 2) using 200 μmol benzoyl‐CoA and 200 mM glycine as substrates [9, 10]. Data given are mean ± standard deviation with *n* = 10 for the wild type and *n* = 5 for the two other samples.

To avoid a liver biopsy for enzyme studies, *GLYAT* wildtype (NM_201648.3) and *GLYAT* mutants c.322C>T: p.(Q108Ter) and c.182A>T: p.(Q61L), known to result in partial GLYAT deficiency [9] (self‐made by site‐directed mutagenesis or obtained from GenScript [Piscataway, NJ, USA]) were overexpressed in *E. coli* origami 2(DE3) cells using a pET‐32a(+) vector [16]. Protein purification via His‐tag was also done according to Schulke and Sass [9], except that HisTrap HP columns (Cytiva #17524701) were used instead of Ni‐TED 2000 columns to isolate GLYAT proteins. Except for the Bradford assay replacing the Lowry method for protein quantitation, protein studies followed the previous descriptions [9]. Western blot analysis showed the absence of GLYAT protein in samples with GLYAT mutant c.322C>T: p.(Q108Ter) and with the vector without insert, while wildtype and *GLYAT* c.182A>T: p.(Q61L) yielded the expected band at 55 kDa for GLYAT carrying a thioredoxin (Trx) tag to enhance protein solubility and His‐tags (Figure 2). GLYAT enzyme activity of GLYAT mutant c.322C>T: p.(Q108Ter) corresponded to that obtained with the empty vector, while the enzyme with the reference sequence (“wild type”) yielded much higher GLYAT activity (Figure 3).

### 2.4 Treatment

Treatment with prednisolone was initiated upon her first hospital admission with a diagnosis of nephrotic syndrome. After finding high plasma glycine, the child was placed on a low‐protein diet and given special milk. Following the discovery of the genetic mutation, a mitochondrial cocktail—consisting of coenzyme Q10, vitamins B1, B2, B6, B12, C, folate, and l‐carnitine—was initiated. Vitamin B5 was also given as a substitute for low CoA levels.

After starting treatment, an improvement in symptoms was noticed in a few days; edema and hypotonia were alleviated, and her visual, verbal, and social interactions became more responsive. Her serum glycine levels normalized within three months. Additionally, total cholesterol and LDL‐C levels decreased, although they remained above normal; therefore, atorvastatin was prescribed for further lipid management.

Unfortunately, due to poor compliance by the parents caused by their divorce and socioeconomic difficulties, the treatment was discontinued by them, causing her symptoms to relapse. A COVID‐19 infection also worsened the patient's condition. She was readmitted with edema and renal insufficiency. Therefore, a pediatric nephrologist reintroduced high‐dose prednisolone. After one month, due to persistent edema, cyclosporine was initiated at a dose of 40 mg/m^2^ for four weeks. Upon the patient's return to the pediatric endocrinology clinic two weeks after starting cyclosporine, the glycine level was measured at 498 μmol/L (normal range: 138–349 μmol/L). Her lipid profile showed elevated total cholesterol of 957 mg/dL, triglycerides of 2598 mg/dL, HDL of 34 mg/dL (normal: 31–73 mg/dL), and LDL of 106 mg/dL. The previous specific treatment regimen was reinstated, leading to the alleviation of symptoms. The serum glycine decreased to 341 μmol/L; thus, cyclosporine was discontinued.

Due to elevated total cholesterol, high LDL, and low HDL levels, atorvastatin and ezetimibe were added to the treatment regimen. Taurine was also prescribed to enhance bile acid conjugation to reduce serum lipids, but this approach was ineffective and was subsequently discontinued.

Cognitive and psychological evaluations indicated improvement, with the score of the Bayley test increasing from 72 to 87. Her IQ test also showed some improvements.

The study was exempt from IRB oversight due to the retrospective data and patient de‐identification. Informed consent was obtained from the patient's guardian.

## 3 Discussion

We present a novel case of GLYAT enzyme deficiency in a patient with developmental delay, autistic features, and locomotor dysfunction. GLYAT plays a crucial role in releasing CoASH from various substrates and maintaining intracellular CoASH levels [11, 12]. CoASH is essential for energy metabolism, and its depletion impairs glucose and lipid metabolism, inhibiting the Krebs cycle [13]. Consequently, GLYAT deficiency may lead to sequestration and depletion of CoASH, limiting energy production and reducing cellular ATP levels. This might explain the patient's developmental delay and movement difficulties, as disruptions in energy production pathways are common in inborn errors of metabolism. Such conditions can weaken skeletal and cardiac muscle function, leading to motor impairment [14]. Accordingly, the patient was prescribed a mitochondrial cocktail, which was followed by an improvement. Pantothenic acid (vitamin B5), a precursor of CoASH [13], was also included in the regimen to address CoASH depletion, which may have contributed to symptom improvement.

Another important function of GLYAT is preventing the accumulation of acyl‐CoAs to toxic levels. When acyl‐CoA levels rise, they can deplete carnitine by serving as substrates for carnitine acyltransferases [15]. Carnitine accepts the transfer of acyl groups from CoASH derivatives, facilitating their excretion and freeing up CoASH [13]. Based on this rationale, carnitine was administered to the patient, which proved effective.

One of the early findings in this patient was mildly elevated glycine levels. It has been proposed that the GLYAT enzyme plays a role in the homeostasis of glycine levels and that the major means of glycine removal from its systemic pool is not by metabolic degradation but by the function of GLYAT through the glycine deportation system [6]. Therefore, GLYAT deficiency may lead to an increased level of glycine due to its decreased removal. Increased serum glycine might partly explain the developmental delay and autistic features because glycine is a major inhibitory neurotransmitter that regulates muscle coordination during movement [16], and acts as a crucial co‐agonist of glutamate at NMDA receptors [17]. Dysfunction of NMDA receptors has been associated with attention disturbances [18], and elevated glycine levels are common in patients with attention deficit disorders [19].

Despite high serum glycine levels, the patient's CSF glycine was within normal limits. It has been previously shown that CSF glycine levels are significantly lower than serum levels, with a plasma‐to‐CSF ratio of approximately 45‐fold [20]. Additionally, in mice injected intraperitoneally with amino acids, there was no uptake of glycine, in contrast to most of the other amino acids [21]. In another study, a 13‐fold increase in plasma glycine levels through a glycine‐fortified diet in animals resulted in only a 2.2‐fold increase in CSF glycine levels. It is well known that CSF glycine levels do not necessarily correlate with those of plasma, and more significant rises in plasma glycine levels would be required to affect its concentration in CSF [22]. Considering the normal levels of glycine in CSF, the neurologic symptoms of the patient might be attributed to the accumulation of xenobiotics or CoASH depletion. GLYAT is crucial for the metabolism of benzoate and salicylate, which are normally present in food sources, are added as preservatives to food, and can also be produced by the gut microbes [23]. Isovaleryl‐CoA is also a substrate for GLYAT and can be conjugated to glycine, forming isovalerylglycine [3]. Glycine supplementation has been shown to be beneficial in the treatment of isovaleric acidemia [2, 24]. Thus, the accumulation of these substrates can participate in the neurologic manifestations. On the other hand, considering the role of the glycine conjugation pathway in maintaining a balance in CoA levels [15], reduced CoASH levels might be another cause of neurologic consequences.

In the patient reported here, to the elevated glycine levels, benzoate, which is typically effective in treating hyperglycinemia, was initially prescribed. However, this treatment worsened her condition and triggered severe seizures. This exacerbation aligns with the defective GLYAT function, critical for metabolizing and excreting benzoic acid. As a result, it seems logical that GLYAT deficiency led to the toxic accumulation of benzoate [11, 25].

Glycine conjugation plays a crucial role in the metabolism of many xenobiotics, primarily occurring in the liver or kidney. If a xenobiotic acyl‐CoA is generated but cannot undergo further metabolism, it accumulates, leading to toxicity, mitochondrial dysfunction, and inhibition of beta‐oxidation [26, 27, 28]. Therefore, kidney dysfunction and nephrotic syndrome might be attributed to the toxic accumulation of these xenobiotics. Glycinuria was due to different mechanisms, including the overflow resulting from the high plasma glycine levels, high activity of the GLYAT enzyme in the kidney, as well as the renal defect that occurred in the patient, which might be due to the toxic accumulation of GLYAT substrates.

CoA is crucial in fat metabolism, including fatty acid synthesis, transport, and degradation. Pantothenic acid supplementation has been shown to reduce total cholesterol, LDL cholesterol, and triglycerides while slightly increasing HDL cholesterol across various types of dyslipidemia, highlighting the relationship between dyslipidemia and CoA [29]. Therefore, the dyslipidemia observed in our patient may be linked to CoA sequestration and the consequent disruption of lipid metabolism.

There is no direct or indirect evidence linking the patient's condition to hypothyroidism, and other etiologies may be involved, especially considering that the patient's mother also had hypothyroidism during pregnancy.

This is the first report of a patient with homozygosity for a GLYAT sequence variant, which fully abolishes the enzyme activities. In contrast, the frequent GLYAT variant p.(Q61L), which accounts for about 12% of the alleles in a Caucasian Afrikaner cohort from South Africa, carries major residue activity [10]. GLYAT haplotype variants with low activities have very low haplotype frequencies and have not been linked to homozygous individuals so far. Therefore, the GLYAT pathway has been reported to be essential for human life [10, 30].

However, in this study, molecular analysis of the proband by WES revealed a homozygous variant (NM_201648.3: c.322C>T) in the *GLYAT*. The frequency of this variant is 0.0000598 (gnomad.broadinstitute.org). In line with *GLYAT* c.322C>T being predicted to cause early truncation of the GLYAT protein (p.(Q108Ter)), our in vitro studies have now shown that no functional GLYAT enzyme is found and that the results with this mutant reflect those obtained after transformation with the vector without insert.

## 4 Conclusion

We describe GLYAT deficiency as a novel inborn error of metabolism in humans, following an autosomal recessive trait of inheritance. Administration of pantothenic acid, l‐carnitine, and a mitochondrial cocktail was effective in alleviating the patient's symptoms.

## Author Contributions

Mona Nourbakhsh, Parvaneh Karimzadeh, Mitra Nourbakhsh, Ali Talea, Elham Pourbakhtyaran, Nakisa Hooman, and Maryam Razzaghy‐Azar were involved in the diagnosis and treatment of the patient. Mohammad Miryounesi Hossein Sadeghi, Mohammad‐Reza Ghasemi, and Nasrin Alipour performed the genetics analysis; Lil Klaas, Daniel Schulke, and Jörn Oliver Sass performed the protein overexpression, purification, and partial characterization; Mitra Nourbakhsh wrote the manuscript, and Jörn Oliver Sass critically revised the final manuscript.

## Ethics Statement

The patient's guardian gave informed consent and publication consent. The ethical approval was waived for this study.

## Consent

All procedures followed were in accordance with the ethical standards of the responsible committee on human experimentation (institutional and national) and with the Helsinki Declaration of 1975, as revised in 2000. Informed consent was obtained from the patient's parents.

## Conflicts of Interest

The authors declare no conflicts of interest.

## Supporting information


**Data S1–S3** Supporting Information.

## Data Availability

Data will be made available from the correspond author upon request.

## References

[1] M. Matsuo , K. Terai , N. Kameda , et al., “Designation of Enzyme Activity of Glycine‐N‐Acyltransferase Family Genes and Depression of Glycine‐N‐Acyltransferase in Human Hepatocellular Carcinoma,” Biochemical and Biophysical Research Communications 420 (2012): 901–906, 10.1016/j.bbrc.2012.03.099. 22475485

[2] Y. R. Mawal and I. A. Qureshi , “Purification to Homogeneity of Mitochondrial Acyl Coa:Glycine n‐Acyltransferase From Human Liver,” Biochemical and Biophysical Research Communications 205 (1994): 1373–1379, 10.1006/bbrc.1994.2817. 7802672

[3] S. Kühn , M. E. Williams , M. Dercksen , J. O. Sass , and R. van der Sluis , “The Glycine N‐Acyltransferases, GLYAT and GLYATL1, Contribute to the Detoxification of Isovaleryl‐CoA ‐ An In‐Silico and in Vitro Validation,” Computational and Structural Biotechnology Journal 21 (2023): 1236–1248, 10.1016/j.csbj.2023.01.041. 36817957 PMC9932296

[4] J. M. Rohwer , C. Schutte , and R. van der Sluis , “Functional Characterisation of Three Glycine N‐Acyltransferase Variants and the Effect on Glycine Conjugation to Benzoyl‐CoA,” International Journal of Molecular Sciences 22 (2021): 22, 10.3390/ijms22063129. PMC8003330 33803916

[5] C. P. S. Badenhorst , E. Erasmus , R. van der Sluis , C. Nortje , and A. A. van Dijk , “A New Perspective on the Importance of Glycine Conjugation in the Metabolism of Aromatic Acids,” Drug Metabolism Reviews 46 (2014): 343–361, 10.3109/03602532.2014.908903. 24754494

[6] D. Beyoğlu and J. R. Idle , “The Glycine Deportation System and Its Pharmacological Consequences,” Pharmacology & Therapeutics 135 (2012): 151–167, 10.1016/j.pharmthera.2012.05.003. 22584143 PMC3665358

[7] N. Ramirez , J. M. Flynn , F. Casalduc , S. Rodriguez , A. S. Cornier , and S. Carlo , “Musculoskeletal Manifestations of Neonatal Nonketotic Hyperglycinemia,” Journal of Children's Orthopaedics 6 (2012): 199–203. 10.1007/s11832-012-0407-1 PMC3400000 23814620

[8] P B, ID A , “Bayley Scales of Infant and Toddler Development,” in StatPearls Treasure Island (StatPearls Publishing, 2025). 33620792

[9] D. Schulke and J. O. Sass , “Frequent Sequence Variants of Human Glycine N‐Acyltransferase (GLYAT) and Inborn Errors of Metabolism,” Biochimie 183 (2021): 30–34, 10.1016/j.biochi.2021.02.002. 33567294

[10] R. van der Sluis , C. P. Badenhorst , F. H. van der Westhuizen , and A. A. van Dijk , “Characterisation of the Influence of Genetic Variations on the Enzyme Activity of a Recombinant Human Glycine N‐Acyltransferase,” Gene 515 (2013): 447–453, 10.1016/j.gene.2012.12.003. 23237781

[11] C. P. Badenhorst , M. Jooste , and A. A. van Dijk , “Enzymatic Characterization and Elucidation of the Catalytic Mechanism of a Recombinant Bovine Glycine N‐Acyltransferase,” Drug Metabolism and Disposition 40 (2012): 346–352, 10.1124/dmd.111.041657. 22071172

[12] T. Sakuma , “Alteration of Urinary Carnitine Profile Induced by Benzoate Administration,” Archives of Disease in Childhood 66 (1991): 873–875, 10.1136/adc.66.7.873. 1863104 PMC1793232

[13] G. A. Mitchell , N. Gauthier , A. Lesimple , S. P. Wang , O. Mamer , and I. Qureshi , “Hereditary and Acquired Diseases of Acyl‐Coenzyme A Metabolism,” Molecular Genetics and Metabolism 94 (2008): 4–15, 10.1016/j.ymgme.2007.12.005. 18337138

[14] A. M. Das , U. Steuerwald , and S. Illsinger , “Inborn Errors of Energy Metabolism Associated With Myopathies,” Journal of Biomedicine & Biotechnology 2010 (2010): 340849, 10.1155/2010/340849. 20589068 PMC2877206

[15] C. P. Badenhorst , R. van der Sluis , E. Erasmus , and A. A. van Dijk , “Glycine Conjugation: Importance in Metabolism, the Role of Glycine N‐Acyltransferase, and Factors That Influence Interindividual Variation,” Expert Opinion on Drug Metabolism & Toxicology 9 (2013): 1139–1153, 10.1517/17425255.2013.796929. 23650932

[16] H. Nishimaru and M. Kakizaki , “The Role of Inhibitory Neurotransmission in Locomotor Circuits of the Developing Mammalian Spinal Cord,” Acta Physiologica (Oxford, England) 197 (2009): 83–97, 10.1111/j.1748-1716.2009.02020.x. 19673737

[17] A. de Bartolomeis , M. Manchia , F. Marmo , L. Vellucci , F. Iasevoli , and A. Barone , “Glycine Signaling in the Framework of Dopamine‐Glutamate Interaction and Postsynaptic Density. Implications for Treatment‐Resistant Schizophrenia,” Frontiers in Psychiatry 11 (2020): 369, 10.3389/fpsyt.2020.00369. 32477178 PMC7240307

[18] J. P. Chang , H. Y. Lane , and G. E. Tsai , “Attention Deficit Hyperactivity Disorder and N‐Methyl‐D‐Aspartate (NMDA) Dysregulation,” Current Pharmaceutical Design 20 (2014): 5180–5185, 10.2174/1381612819666140110115227. 24410567

[19] K. F. Holton , J. Johnstone , and J. T. Nigg , “The Association of Dietary Glycine and Glutamate With ADHD,” FASEB Journal 30, no. Suppl 1 (2016): 679.8, 10.1096/fasebj.30.1_supplement.679.8.

[20] K. Iijima , S. Takase , K. Tsumuraya , M. Endo , and K. Itahara , “Changes in Free Amino Acids of Cerebrospinal Fluid and Plasma in Various Neurological Diseases,” Tohoku Journal of Experimental Medicine 126 (1978): 133–150, 10.1620/tjem.126.133. 715764

[21] L. Battistin , A. Grynbaum , and A. Lajtha , “The Uptake of Various Amino Acids by the Mouse Brainin Vivo,” Brain Research 29 (1971): 85–99, 10.1016/0006-8993(71)90419-7. 5564265

[22] S. Scholl‐Bürgi , S. H. Korman , D. A. Applegarth , et al., “The Relation of Cerebrospinal Fluid and Plasma Glycine Levels in Propionic Acidaemia, a 'ketotic Hyperglycinaemia',” Journal of Inherited Metabolic Disease 31 (2008): 395–398, 10.1007/s10545-008-0796-y. 18392751

[23] A. Del Olmo , J. Calzada , and M. Nuñez , “Benzoic Acid and Its Derivatives as Naturally Occurring Compounds in Foods and as Additives: Uses, Exposure, and Controversy,” Critical Reviews in Food Science and Nutrition 57 (2017): 3084–3103, 10.1080/10408398.2015.1087964. 26587821

[24] M. Naglak , R. Salvo , K. Madsen , P. Dembure , and L. Elsas , “The Treatment of Isovaleric Acidemia With Glycine Supplement,” Pediatric Research 24 (1988): 9–13, 10.1203/00006450-198807000-00004. 3137519

[25] M. Kelley and D. A. Vessey , “Characterization of the Acyl‐CoA:Amino Acid N‐Acyltransferases From Primate Liver Mitochondria,” Journal of Biochemical Toxicology 9 (1994): 153–158, 10.1002/jbt.2570090307. 7983681

[26] K. M. Knights and J. O. Miners , “Amino Acid Conjugation: A Novel Route of Xenobiotic Carboxylic Acid Metabolism in Man,” In Encyclopedia of Drug Metabolism and Interactions (Wiley, 2012), 1–16.

[27] K. W. Yao , L. F. Mao , M. J. Luo , and H. Schulz , “The Relationship Between Mitochondrial Activation and Toxicity of Some Substituted Carboxylic Acids,” Chemico‐Biological Interactions 90 (1994): 225–234, 10.1016/0009-2797(94)90012-4. 8168171

[28] D. Galvan , N. Green , and F. Danesh , “The Hallmarks of Mitochondrial Dysfunction in Chronic Kidney Disease,” Kidney International 92 (2017): 1051–1057, 10.1016/j.kint.2017.05.034. 28893420 PMC5667560

[29] Z. Horváth and L. Vecsei , “Treatment With Pantethine,” Complementary Health Practice Review 16 (2011): 21–28, 10.1177/1533210110392944.

[30] R. van der Sluis , C. P. Badenhorst , E. Erasmus , E. van Dyk , F. H. van der Westhuizen , and A. A. Van Dijk , “Conservation of the Coding Regions of the Glycine N‐Acyltransferase Gene Further Suggests That Glycine Conjugation Is an Essential Detoxification Pathway,” Gene 571 (2015): 126–134. 26149650 10.1016/j.gene.2015.06.081

